# Phytochemical Approach and Bioanalytical Strategy to Develop Chaperone-Based Medications

**DOI:** 10.2174/1874091X00802010044

**Published:** 2008-04-29

**Authors:** Bernd Kastenholz

**Affiliations:** Aachen City Region, North Rhine-Westphalia, Eschweiler (Rhld.) 52249, Germany

**Keywords:** Phytochaperones, biofluids, Alzheimer’s disease, medicinal plant extracts, CCS, Ginkgo biloba, SOD, molecular farming, GPC, metal cofactors, homeostasis, QPNC-PAGE

## Abstract

Currently, no pharmaceuticals for the etiological treatment of degenerative protein-misfolding diseases (e.g., ALS, Alzheimer’s or prion diseases) are commercially available. In this technical note theoretical considerations and practical approaches concerning the development of chaperone-based medications from medicinal plants (e.g., *Ginkgo biloba*) are reviewed and discussed in detail. Phytochaperones and other agents isolated from medicinal plants are proposed to serve as the general basis of drug development in protein-misfolding diseases.

## INTRODUCTION 

“Nature is, after all, the only book that offers important content on every page” (Johann Wolfgang von Goethe, 1749-1832). This aphorism of the famous poet and natural scientist reflects that we should consider all natural sources for answers to problems concerning human health and welfare. Medicinal plants, for example, may be important options for developing drugs for the treatment of different diseases.

Despite enormous economical, technical and scientific progress in past years, fundamental development of effective treatments for several progressive degenerative and neurodegenerative diseases (e.g., amyotrophic lateral sclerosis (ALS), Alzheimer’s disease (AD), or Parkinson’s disease, *etc*.) is in its infancy. Many of these diseases are caused by the misfolding of one or more important proteins.

It is a well-known fact that chemical and pharmacological chaperones have been found to be effective in preventing the misfolding of different disease-causing proteins. However, many of these compounds are highly toxic, reveal a lack of specificity, or have unknown mechanisms of action *in vivo*. This technical note introduces a new class of pharmacologically active proteins, namely the *(metallo-)* *phytochaperones*, as possibly key molecules for the etiological treatment of protein-misfolding diseases (PMD).

## MEDICINAL PLANTS AND PROTEIN-MISFOLDING DISEASES

Drug discovery from medicinal plants is a challenging field because it involves a multifaceted approach combining botanical, phytochemical, biological, and analytical techniques [1-6]. Well-known plants used in Traditional Chinese Medicine (TCM), Japanese, Ayurvedic and European Medicine relevant to the management of AD and other cognitive disorders are listed in Table **1** [1, 16, 17, 25, 31]. For

example, standardized plant extracts from green leaves of the *Ginkgo biloba* tree are generally accepted in the treatment of AD [2, 3,9, 16, 19, 31]. Through the antioxidant properties of their *flavonoids* these extracts may be able to protect hippocampal cells against toxic effects induced by amyloid ß (Aß) peptides [2]. An increase in the activity of the antioxidant enzymes, *catalase* and *superoxide dismutase* (SOD) were further observed in rats treated with EGb 761 *Ginkgo* extract [3]. Another plant used in Ayurvedic medicine, termed *Bacopa monniera,* reduces Aß deposits in brains of AD model animals [7].

AD and many other neurodegenerative diseases are associated with disturbances of metal ion metabolisms and oxidative stresses postulated to be a downstream effect of abnormal Aß - metal ion interactions [6, 14, 20, 21, 24, 26]. Therefore, the metal ion homeostasis in a cell is strictly regulated by metallochaperones and other biomolecules (e.g., metallothioneins). For example, copper chaperones for superoxide dismutase (CCS) are essential metalloproteins for protecting and guiding copper ions to superoxide dismutase (SOD). Specific protein-protein interactions activate SOD by incorporating a Cu^+^ ion. As properly folded SOD molecules are very important antioxidants, these metal species contribute to a decreasing oxidative stress in cells [15, 20, 21, 28]. Thus, metal chelators and antioxidants may be therapeutic against neurodegenerative diseases [5, 6, 9, 14, 24]. (See especially the insightful review by Rochet [32].)

Despite several therapeutic approaches, no preventive measure or effective treatment for PMD, especially Alzheimer’s disease, is currently available [26]. Furthermore, vast majorities of psychoactive drugs are not natural products or are not derived from bioactive constituents of medicinal plants [25]. This has led some researchers to recommend the use of natural plant extracts in seeking possible protective agents of brain aging [2] and dementia therapy [31].

Plant extracts are multicomponent mixtures consisting of bioactive main and secondary plant compounds which may interact with each other in a synergistic manner [8, 12, 19]. Drying and storing of medicinal plants are critical steps in the production processes of natural extracts and phytomedicines because the chemical stability of the bioactive ingredients may be adversely affected by the formation of unwanted artefacts [4]. As nature is the best combinatorial chemist and possibly has answers to all diseases of mankind [17] it is assumed that pharmacologically active ingredients in addition to the well-known plant flavonoids and terpenoids, namely proteins and enzymes, could be isolated and identified in medicinal plants for the effective treatment of several PMD (see Table **1**).

A majority of PMD are considered to be caused primarily by an imbalance between pro-oxidant and antioxidant homeostasis [34]. An ideal therapeutic drug to dissolve Aß amyloid in AD, for example, would involve a compound selective for Cu^1+^, Zn^2+^ and Fe^3+^, but that does not sequester Mg^2+^ and Ca^2+^ [6]. For example, Cu chaperones are a ubiquitous class of proteins that play a significant role in both Cu delivery and cellular protection against copper exposure under normal metabolic conditions by delivering *and* binding metal ions [15, 28]. Therefore, bioactive Cu chaperones may be the basis for developing novel lead molecules in the treatment of PMD.

It is a well-known fact that improperly folded copper chaperones for superoxide dismutase (CCS) may play an important role in the etiology of AD and other PMD. Therefore, the dysregulation of metal ion homeostasis and severe oxidative stresses in bioorganisms may occur in these diseases [20, 21]. Furthermore, under non-physiological conditions a reduced enzyme activity of SOD and apo-SOD (apoenzymes) can be detected and quantified in the blood of animals [36]. The apoenzymes are referred to as unfolded molecules. Therefore, it may be a necessary and helpful therapeutic approach to balance the metal ion homeostasis by activating unfolded SOD in blood of diseased bioorganisms. For these purposes, exogenous plant copper chaperones for SOD (pCCS) isolated from medicinal plants may be the lead molecules for an effective treatment of PMD. The pCCS activators may be able to recover the balance between pro-oxidant and antioxidant homeostasis of bioorganisms by copper ion transfer affecting the mechanism and speed of folding for the rapid achievement of the bioactive 3D conformation of human SOD (hSOD) *and* by binding uncomplexed metal ions (e.g., Cu^1+^, Zn^2+^ or Fe^3+^) in blood or other biofluids of living organisms.

## DEVELOPMENT OF CHAPERONE-BASED MEDICATIONS

For developing chaperone-based medications from medicinal plants the following procedures could be very promising. For these purposes, properly and improperly folded copper cofactor-containing chaperones for superoxide dismutase present in blood samples of AD patients and probands have to be purified and their structures elucidated. Improperly folded and bioactive metallochaperone proteins present in diseased or healthy blood can be resolved in electrophergrams due to their different isoelectric points [18, 20, 21]. Bioactive and inactive metalloproteins can also be isolated and quantified in other organisms, e.g., model plants by using the same methods [20-23]. In Fig. (**1**) the basic investigational steps of selected protein-protein interactions and metalloprotein detection procedures in complex biological systems are schematically presented.

By incubating clinical biofluids (*e.g*., whole blood) with medicinal plant extracts (*e.g*., *Ginkgo biloba*), specific apoenzymes in a pathological blood sample (apo-SOD) might fold into their native conformation due to specific protein-protein interactions. Human SOD is a biomacromolecule with a molecular mass of about 32 kDa and might interact with the investigated plant CCS provided that pCCS has a similar molecular mass and structure and function compared to human CCS. The respective physiological effects can be studied using the workflow schemes of Fig. (**1**).

Plant extracts may be obtained by homogenising leaves of medicinal plants in liquid nitrogen. Pulverized samples may be stored above liquid nitrogen or extracted directly. Medicinal plant extracts are prepared under non-denaturing conditions by using a buffer such as 20 mM Tris-HCl, pH 7.2. Plant material and buffer solution may be homogenised in a ratio of 1:10 (w/w). After centrifugation of the homogenate the resultant supernatant is used for merging plant extract and blood. The incubation time is extended to a maximum of about 15 to 60 minutes at 4° C to avoid uncontrolled proteolytic processes, protein precipitation, and destabilization of metal cofactor-containing proteins in this very complex system consisting of plant and human matrices. Next, an aliquot of the protein mixture is chromatographed on a Sephadex G-50 SF size-exclusion column.

Specific metal cofactor-containing proteins CCS and SOD have been found in a narrow peak (MW ≥ 30 kDa) as shown in Fig. (**2**).

After chromatography, fractions with the highest Cu concentration can be separated by an orthogonal procedure to resolve the peak components. A procedure that has shown promise for this kind of secondary fractionation is quantitative preparative native continuous polyacrylamide gel electrophoresis (QPNC-PAGE) [21]. With QPNC-PAGE, physiological amounts of properly folded hSOD and pCCS may be isolated in a few specific PAGE fractions. Furthermore, the respective Cu cofactors of these biomolecules can be detected in the resultant electropherogram by mass-spectrometric methods. The complementary QPNC-PAGE parameters of CCS and SOD have been listed in various articles or protocols [20-23, 33]. The ratio of peak areas of the copper species may indicate whether certain medicinal plants contain bioactive pCCS upon comparison of untreated and treated blood from AD patients.

In order to develop chaperone-based medications from medicinal plants, highly purified pCCS may be further isolated and identified after the QPNC-PAGE run using a combination of 2-D PAGE (2-DE) followed by matrix-assisted laser desorption ionization time-of-flight mass spectrometry (MALDI-TOF) and bioinformatics (Fig. (**3**)). The limitations of current proteomics technologies as related to MALDI-TOF and 2-DE are reviewed in [13]. An approach for identifying a high molecular mass metal protein in the model plant *Arabidopsis thaliana* by using these efficient methods is presented in [35].

The amino acid sequences of identified bioactive metallochaperone proteins in medicinal plants will provide the DNA sequence information necessary for their effective production by “molecular farming” techniques (Fig. (**3**)). Plant molecular farming is a challenging, new and promising technique using transgenic plants to produce foreign proteins as pharmaceutical ingredients. For these purposes plant growth and metabolism has to be optimized under standardized conditions in order to maximize protein concentrations in roots and shoots [29]. It is important to mention that this method is an alternative to the microbial expression systems enabling the correct folding of recombinant proteins [11, 30]. Of course, many other clinical and pharmaceutical approaches as already described in literature [1-36] have to be used for development of chaperone-based medications.

## CONCLUSIONS AND OUTLOOK

In this technical note, it is proposed that metallochaperones from medicinal plants may provide treatments for protein misfolding diseases (e.g., Alzheimer’s disease) and analytical steps are presented by which to test this conjecture. In particular, plant copper chaperones for superoxide dismutase (pCCS) could very well be the sources for the etiological treatment of protein-misfolding diseases, because pCCS may have the ability to activate human apo-superoxide dismutase (hSOD) in biofluids. The interaction between pCCS and apo-SOD in human beings and animals may be important for recovering the metal ion homeostasis and balance between pro-oxidative and antioxidative processes in the cells of these organisms. Therefore, this approach could help to prevent or minimize abnormal protein-misfolding processes and subsequent oxidative stresses occuring in bioorganisms.

In addition to the well-known medicinal plants, e.g., *Ginkgo biloba*, other “living fossils” should be evaluated with respect to chelating and antioxidative properties in cells concerning the effective treatment of protein-misfolding diseases. For example, giant trees known as California Redwoods (e.g., *Sequoia sempervirens*), might be the natural sources of active metallochaperones or other important metal species.

## Figures and Tables

**Fig. (1):**
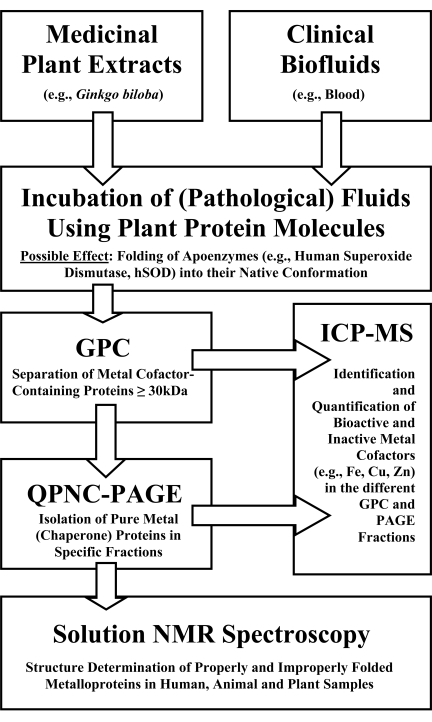
Workflow schemes in metalloproteomics and interactomics.

**Fig. (2):**
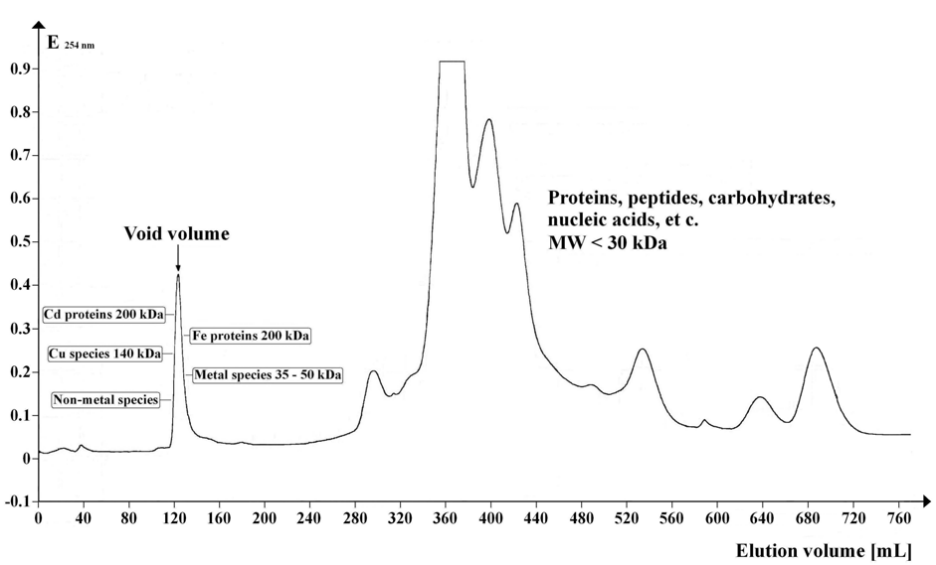
Chromatogram showing the UV absorption profile (λ = 254 nm) of Arabidopsis supernatant separated on Sephadex G-50 Superfine. Gel volume: 500 mL; column length: 700 mm; column diameter: 30 mm; eluent flow rate: 12 mL / hr; fraction volume: 8.0 mL; number of fractions: 95; sample volume: 5 mL; separation temperature: 4 °C; elution buffer: 20 mM Tris-HCl, 1 mM NaN3; pH 8.0. The peripheral tools used for preparative native GPC are listed in [23, 35]. The denoted molecular weights of the detected metal compounds (MW ≥ 30 kDa) are approximated values. Metal cofactors eluted in the range of the void volume (120 to 140 mL) of this method were identified and quanti-fied by ICP-MS or GF-AAS [23, 35].

**Fig. (3):**
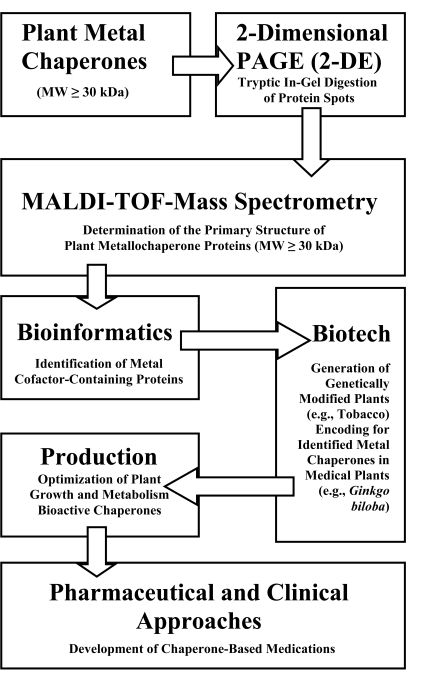
Workflow schemes in metalloproteomics and plant molecular farm-ing

**Table 1:** Traditional Medicinal Plants Used for Cognitive Disorders

Medicinal Plant	Uses, Pharmaceutical and Clinical Effects
*Centella asiatica* L	Strengthens nervous function and memory, enhancement of cholinergic activity and thus, cognitive function.
*Ginkgo biloba*L	Improvement of memory loss associated with blood circulation abnormalties, favourable effects on neuronal cell metabolism, antioxidant activity, neuroprotective against β-amyloid toxicity *in vitro*.
*Melissa officinalis* L	Treatment of depression, hysteria and nervous insomnia, shows antioxidant effects.
*Polygala tenuifolia*Willd	Used in TCM as a cardiotonic and cerebrotonic, as a sedative and tranquillizer, and for amnesia, forgetfulness, neuritis, nightmares and insomnia.
*Salvia lavandulaefolia*Vahl. *Salvia officinalis* L *Salvia miltiorrhiza* Bung	Cholinesterase inhibition, antioxidant and oestrogenic activities *in vitro*. Treatment of blood circulation disorders, insomnia, neurasthenia and alleviation of inflammation.
*Withania somnifera*(L)Dun	Important herb in Ayurvedic medicine, treatment of inflammatory conditions, such as arthritis.
